# VineLiDAR: High-resolution UAV-LiDAR vineyard dataset acquired over two years in northern Spain

**DOI:** 10.1016/j.dib.2023.109686

**Published:** 2023-10-14

**Authors:** Sergio Vélez, Mar Ariza-Sentís, João Valente

**Affiliations:** Information Technology Group, Wageningen University & Research, 6708 PB Wageningen, the Netherlands

**Keywords:** Viticulture, Precision agriculture, LiDAR, Drone, Path planning, Woody crops, Photogrammetry, Structure From Motion (SfM)

## Abstract

LiDAR (Light Detection and Ranging) technology's precision in data collection has gained immense traction in the field of remote sensing, particularly in Precision Agriculture using Unmanned Aerial Vehicles (UAVs). To fulfill the pressing need for public UAV LiDAR datasets in the domain of Agricultural Sciences, especially for woody crops such as vineyards, this study presents an extensive dataset of LiDAR data collected from vineyards in northern Spain. The DJI M300 multi-rotor platform, equipped with a DJI Zenmuse L1 LiDAR sensor, conducted UAV flights at 20, 30, and 50 meters above ground level (AGL) across two vineyards during three development stages in 2021 and 2022. This dataset is composed of ten high-density 3D LiDAR point clouds stored in .laz format with embedded RGB information in each point. It provides insights into vineyard morphology and development, thereby aiding in the optimization of vineyard management strategies. Furthermore, it serves as a valuable tool for agricultural robotics, offering comprehensive terrain information for developing efficient flight paths and navigation algorithms. Finally, it serves as a reliable “ground truth” dataset to validate satellite-derived models, facilitating the creation of highly accurate digital elevation models (DEMs) and other derived models.

Specifications Table

**Table:** 

Subject	Agricultural Sciences, Agronomy and Crop Science
Specific subject area	LiDAR data for Precision Agriculture using UAVs
Type of data	LiDAR
How the data were acquired	Aerial Platform: DJI M300 multi-rotor platformFlight speed: 4 m/s Side overlap: 50% Frontal overlap: 80%LiDAR Sensor: DJI Zenmuse L1Sensor characteristics: Point Rate: Single return: max. 240,000 pts/s; Multiple return: max. 480,000 pts/s. Real-time Point Cloud RGB Coloring. RGB Mapping Camera Effective Pixels: 20 MP. Lidar Ranging Accuracy (RMS 1σ): 3 cm @ 100 m.Flights were conducted during the years 2021 and 2022. Two different vineyards were flown over (B9 and B7). Three data capture phases were carried out:First Phase (September 16, 2021) • Vineyard B9: 30 meters AGL • Vineyard B9: 50 meters AGL Second Phase (July 14, 2022) • Vineyard B7: 20 meters AGL • Vineyard B9: 20 meters AGL • Vineyard B7: 30 meters AGL • Vineyard B9: 30 meters AGL Third Phase (September 8, 2022) • Vineyard B7: 20 meters AGL • Vineyard B9: 20 meters AGL • Vineyard B7: 30 meters AGL • Vineyard B9: 30 meters AGL
Data format	LiDAR RGB point clouds in .laz format
Description of data collection	On September 16, 2021, July 14, 2022, and September 8, 2022, UAV DJI M300 flights were conducted to collect LiDAR data from two vineyards at 20, 30, and 50 meters above ground level (AGL). The flights were programmed to be autonomous, adhering to DJI manufacturer instructions, and utilized RTK (Real-Time Kinematic) technology for precise positioning and accurate navigation. The dataset comprises ten 3D point clouds, with RGB information embedded in each point, and the data were stored in .laz format.
Data source location	Institution: Wageningen University & Research City/Town/Region: Tomiño, Pontevedra, Galicia Country: Spain Vineyard coordinates: Vineyard B7, X: 517186.7, Y: 4645072.3; Vineyard B9, X: 516987.9, Y: 4644817.7 (WGS 84 / UTM zone 29N, EPSG:32629).
Data accessibility	Repository name: Zenodo Data identification number: https://doi.org/10.5281/zenodo.8113105 Direct URL to data: https://zenodo.org/record/8113105

## 1 Value of the Data

• The data provides invaluable insights for professionals and researchers in the field of Agricultural Sciences focusing on the application of LiDAR data for Precision Agriculture using UAVs.
• The dataset captures the specific features of vineyards at different altitudes, allowing detailed 3D reconstruction and a comprehensive understanding of vineyard morphology, which can further aid in the optimization of vineyard management strategies.
• The dataset, acquired over multiple phases in 2021 and 2022, provides a temporal view of vineyard development and changes, which can be used as “ground truth” for satellite-derived models or digital-twin development.
• For agricultural robotics with UAVs or UGVs, the dataset can play a pivotal role because it provides comprehensive terrain information that can assist in the development of efficient flight paths and navigation algorithms in agricultural environments.
• The LiDAR data offers high-density information, permitting the creation of very accurate derived models such as realistic and accurate digital elevation models (DEMs).

## 2 Objective

In recent times, LiDAR (Light Detection and Ranging) technology has become a hot topic in remote sensing due to its ability to deliver highly detailed data [1]. There are already studies showing that UAV-based LiDAR can effectively monitor crop changes [2] and provide efficient tracking of biomass and nitrogen uptake [3]. Additionally, the use of 3D point cloud applications in vineyards has been demonstrated to effectively estimate pruning weight [4], detect vineyards, evaluate vine-rows features [5], and generate accurate digital surface models (DSMs) that aid in creating digital terrain models (DTMs) and Canopy Height Models (CHM) for canopy management [6] and disease detection [7]. On the other hand, there is a growing interest in estimating vineyard parameters from satellite imagery like Sentinel 2 [8,9]. However, to accomplish this, precise ground truth is needed, and UAV data can be used to validate other satellite remote sensing data [10]. To achieve this, high-quality and accurate data, like that provided by LiDAR, is recommended. Thus, UAV LiDAR technology emerges as a promising instrument for precision agriculture with a wide array of applications. However, it is important to note that to acquire LiDAR data, one needs LiDAR sensors which come at a high cost. A solution to this challenge for researchers is the utilization of public UAV LiDAR datasets. But agricultural datasets, particularly those related to woody crops or vineyards, are rare.

Consequently, the main objective of this work is to fill the gap in LiDAR UAV datasets for woody crops, more specifically in vineyards, and to gather comprehensive LiDAR data of vineyards from UAV flights at different altitudes, thus providing in-depth information beneficial for Agricultural Sciences and Precision Agriculture. The data will 1) facilitate a nuanced understanding of vineyard morphology through detailed 3D reconstruction, 2) offer a temporal perspective on vineyard development by presenting data from multiple phases in 2021 and 2022, 3) cater to the needs of agricultural robotics by providing valuable terrain information for flight paths and navigation algorithm optimization, and 4) offer a set of “ground truth” data for satellite-derived models. Utilizing a DJI M300 multi-rotor platform equipped with a DJI Zenmuse L1 LiDAR sensor, autonomous flights were conducted across two vineyards at 20, 30, and 50 meters AGL. The dense and high-quality LiDAR data, stored in .laz format with embedded RGB information, may also be used for the generation of accurate digital elevation models (DEMs) and other derived models.

This study highlights the importance of datasets in applied sciences, particularly in Precision Agriculture. In order to enhance the potential applications of this LiDAR dataset for either Precision Agriculture or Precision Viticulture, this dataset could be combined with previously available datasets that include RGB videos and multispectral images from the same vineyard [11,12]

## 3 Data Description

**Fig. 1:**
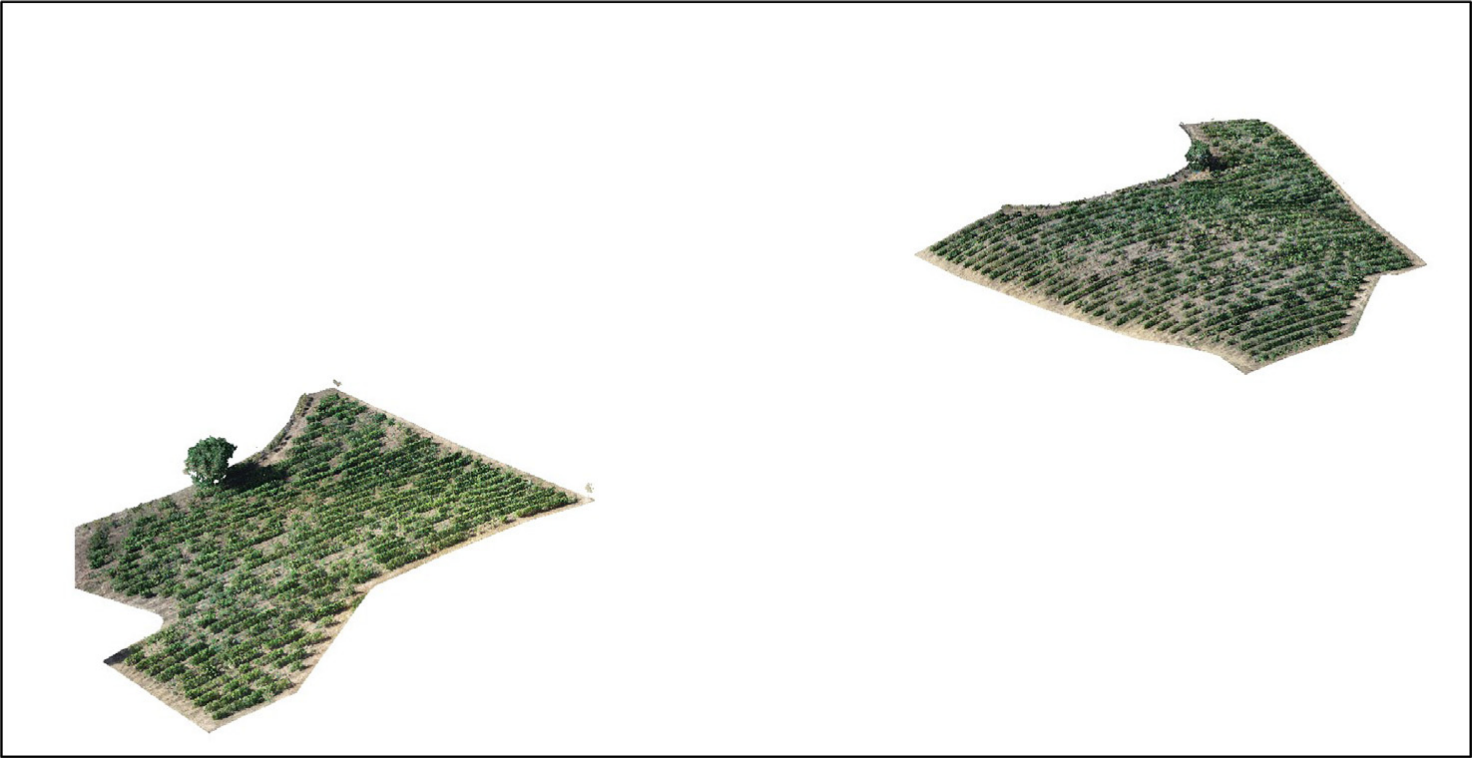
3D view of the vineyards.

This study presents ‘VineLiDAR’, a comprehensive dataset derived from vineyards, which consists of high-density LiDAR 3D point clouds with embedded RGB data (Fig. 1). Providing a spatiotemporal representation of vineyard morphology, the dataset exhibits considerable potential for precision agriculture, particularly in vineyard management, creating ground truth for satellite-derived models or digital twins, and streamlining navigation for agricultural robotics.

The data were captured in three distinct phases on September 16, 2021, July 14, 2022, and September 8, 2022 (Table 1). File names follow the structure “date_FLEXIGROBOTS_sensor_PRO_AGLheight_speed_vineyard.laz,” with an example being “20210916_FLEXIGROBOTS_L1_PRO_30M_4MS_B9.laz,” which corresponds to a UAV flight that took place on September 16, 2021, at an altitude of 30 meters over vineyard B9.

**Table 1:** Characteristics of the flights. The flights were conducted during the years 2021 and 2022. Three data capture phases were carried out. AGL: above ground level.

Phase	Date	Time (UTC)	Vineyard	Altitude AGL	Flight ID
First	September 16, 2021	17:15	B9	30 meters	1
First	September 16, 2021	17:00	B9	50 meters	2
Second	July 14, 2022	17:45	B7	20 meters	3
Second	July 14, 2022	17:00	B9	20 meters	4
Second	July 14, 2022	17:30	B7	30 meters	5
Second	July 14, 2022	16:45	B9	30 meters	6
Third	September 8, 2022	15:45	B7	20 meters	7
Third	September 8, 2022	15:30	B9	20 meters	8
Third	September 8, 2022	15:00	B7	30 meters	9
Third	September 8, 2022	15:15	B9	30 meters	10

**Fig. 2:**
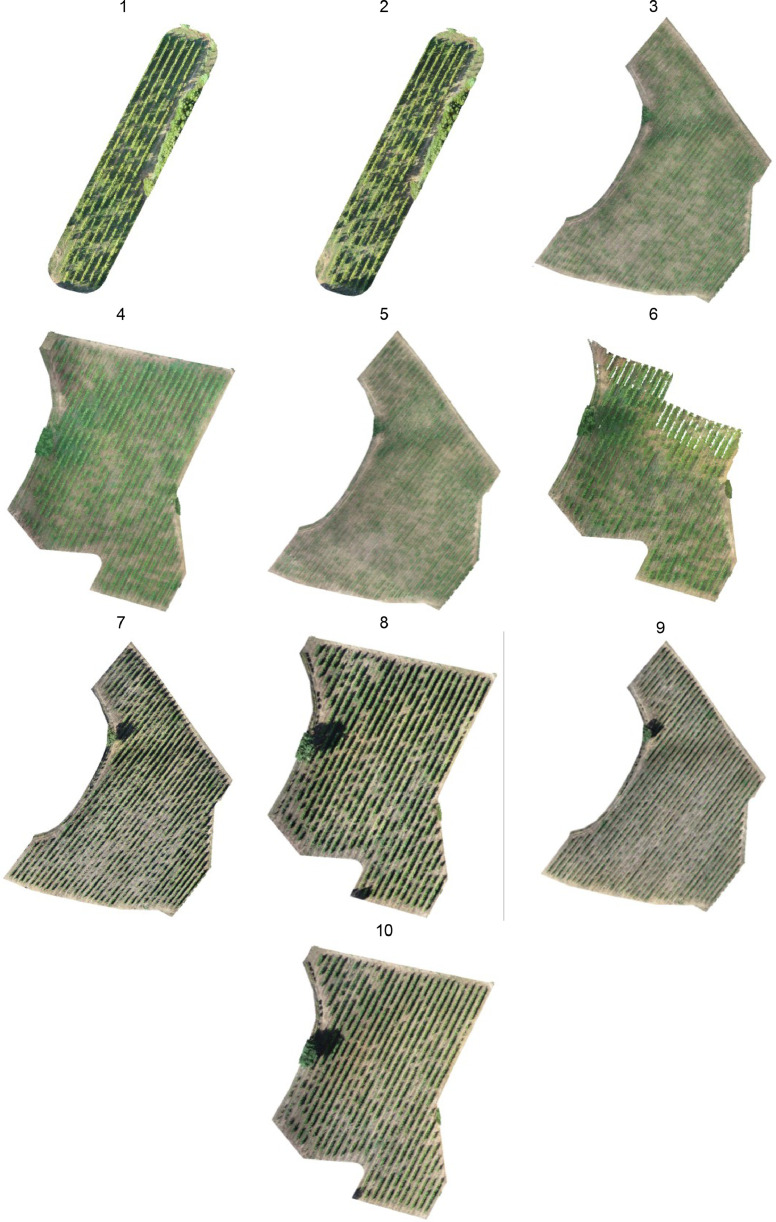
LiDAR point clouds of vineyards. The number corresponds to the Flight ID.

UAV flights were carried out at 20, 30, and 50 meters above ground level (AGL) to collect LiDAR data from two vineyards. The 2021 flights focused primarily on the eastern part of the vineyard. The UAVs were programmed for autonomous flight, adhering to DJI's manufacturer instructions, and used RTK (Real-Time Kinematic) technology to ensure precision in positioning and navigation. The dataset consists of ten LiDAR 3D point clouds (Fig. 2), each with embedded RGB information, and the data is stored in .laz format (Table 2). The flights were conducted in the afternoon, which can affect the RGB information due to the effect of sunlight, causing overexposure or shadowing. However, unlike photogrammetry methods that rely on visible light, LiDAR technology uses pulses of laser light to measure distances, and thus is less directly impacted by sunlight conditions. Therefore, for the LiDAR point cloud data itself, sunlight has minimal impact.

**Table 2:** File size and number of points per LiDAR point cloud.

Flight ID	File name	File size (MB)	Number of points
1	20210916_FLEXIGROBOTS_L1_PRO_30M_4MS_B9.laz	114.7	15,166,707
2	20210916_FLEXIGROBOTS_L1_PRO_50M_4MS_B9.laz	75.7	9,597,830
3	20220714_FLEXIGROBOTS_L1_PRO_20M_4MS_B7.laz	557	71,389,912
4	20220714_FLEXIGROBOTS_L1_PRO_20M_4MS_B9.laz	373.9	46,787,138
5	20220714_FLEXIGROBOTS_L1_PRO_30M_4MS_B7.laz	388.5	47,893,588
6	20220714_FLEXIGROBOTS_L1_PRO_30M_4MS_B9.laz	133	16,058,341
7	20220908_FLEXIGROBOTS_L1_PRO_20M_4MS_B7.laz	528.7	67,522,231
8	20220908_FLEXIGROBOTS_L1_PRO_20M_4MS_B9.laz	247.2	30,544,937
9	20220908_FLEXIGROBOTS_L1_PRO_30M_4MS_B7.laz	250.5	30,973,711
10	20220908_FLEXIGROBOTS_L1_PRO_30M_4MS_B9.laz	172.9	20,699,135

## 4 Experimental Design, Materials and Methods

**Fig. 3:**
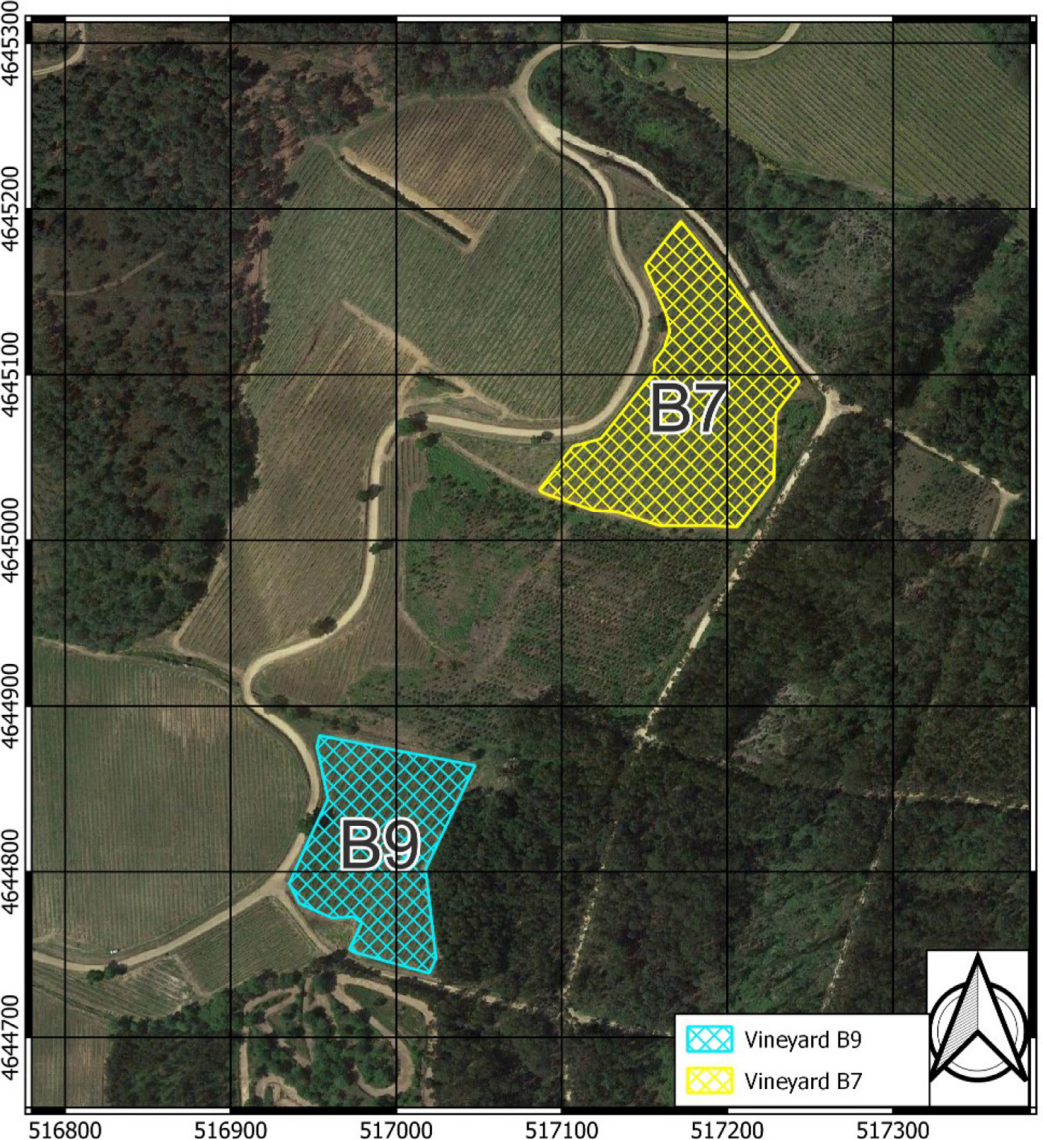
Vineyard locations in Tomiño, Pontevedra, Spain. Coordinates: Vineyard B7, X: 517186.7, Y: 4645072.3; Vineyard B9, X: 516987.9, Y: 4644817.7 (WGS 84 / UTM zone 29N, EPSG:32629).

**Fig. 4:**
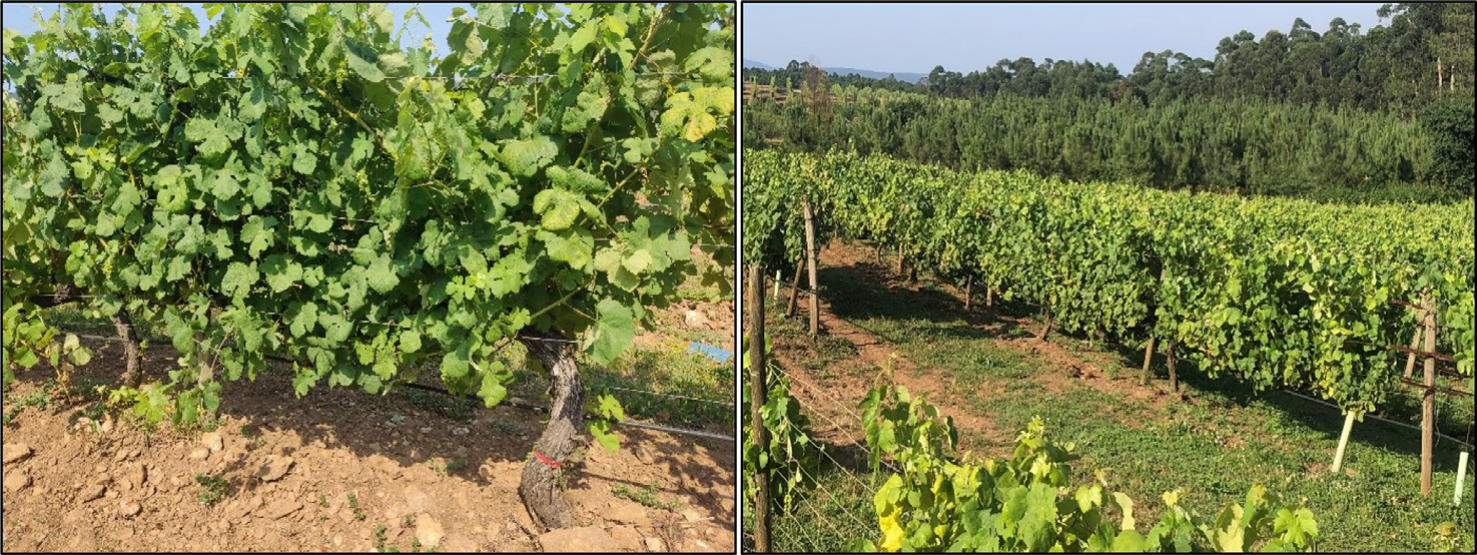
Left: Plants were trained in vertical shoot position (VSP). Right: Spontaneous vegetation species grew as cover crops.

The dataset was obtained from three flight campaigns carried out on September 16, 2021, July 14, 2022, and September 8, 2022. These months were selected to monitor the state of the plants during their vegetative growth phase (July) and to monitor the plants at the critical time of harvest (September). Flights were conducted at altitudes of 20, 30, and 50 meters above ground level (AGL) over two commercial vineyards *Vitis vinifera* cv. Loureiro, designated as B7 and B9 (Fig. 3). The vineyards were property of 'Bodegas Terras Gauda S.A.', and were located in Tomiño, Pontevedra, within the region of Galicia, Spain (Coordinates in WGS 84 / UTM zone 29N, EPSG:32629: Vineyard B7, X: 517186.7, Y: 4645072.3; Vineyard B9, X: 516987.9, Y: 4644817.7). Plants were planted with a NE-SW orientation and were trained in vertical shoot position (VSP) and spontaneous vegetation species grew within the rows as cover crops (Fig. 4).

**Fig. 5:**
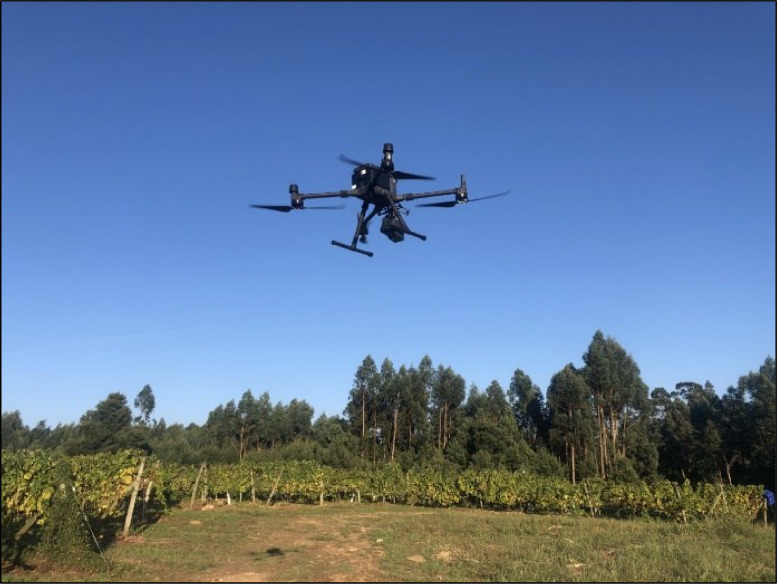
Unmanned Aerial System (UAS) consisting of a DJI M300 UAV equipped with a DJI Zenmuse L1 LiDAR.

**Fig. 6:**
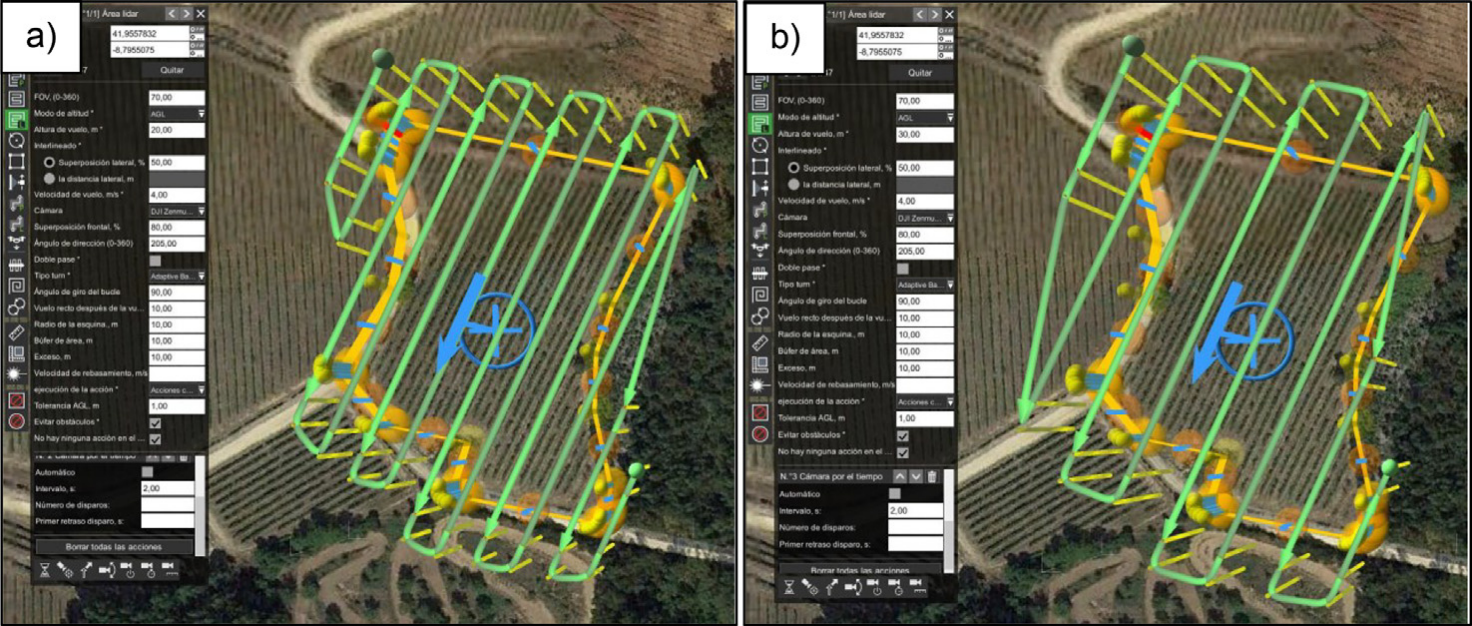
Flight survey paths for LiDAR at (a) 20 m height and (b) 30 m height

The flights were conducted using an Unmanned Aerial System (UAS, Fig. 5) consisting of a DJI M300 multi-rotor platform UAV equipped with a DJI Zenmuse L1 LiDAR sensor (DJI Sciences and Technologies Ltd., Shenzhen, Guangdong, China). Flight planning and execution were done following the vineyard rows and using the combination of DJI PILOT 2 + UgCS software. For each flight, the planned flight speed was 4 m/s, the side overlap was 50%, the frontal overlap was 80%, and the UAV was programmed for autonomous operations with RTK (Real-Time Kinematic) technology for precision navigation (Fig. 6).

The LiDAR sensor features a point rate of a maximum of 240,000 points per second for a single return and 480,000 points per second for multiple returns, and an RGB mapping camera with effective pixels of 20 MP. Table 3 shows the key specifications for the Zenmuse L1 Device, according to the information provided by the manufacturer [13].

**Table 3:** Key specifications for the Zenmuse L1 device.

Feature	Specification
Dimensions	152×110×169 mm
Weight	930±10 g
Power	Typical: 30 W; Max: 60 W
Supported Aircraft	Matrice 300 RTK
Detection Range	450 m @ 80% reflectivity, 0 klx; 190 m @ 10% reflectivity, 100 klx
Point Rate	Single return: max. 240,000 pts/s; Multiple return: max. 480,000 pts/s
System Accuracy (RMS 1σ)1	Horizontal: 10 cm @ 50 m; Vertical: 5 cm @ 50 m
Real-time Point Cloud Coloring Modes	Reflectivity, Height, Distance, RGB
Lidar Ranging Accuracy (RMS 1σ)2	3 cm @ 100 m
IMU Update Frequency	200 Hz
Angular Velocity Meter Range	±2000 dps
Auxiliary Positioning Vision Sensor Resolution	1280×960
Auxiliary Positioning Vision Sensor FOV	95°
RGB Mapping Camera Effective Pixels	20 MP
RGB Mapping Camera Photo Size	5472×3078 (16:9), 4864×3648 (4:3), 5472×3648 (3:2)
RGB Mapping Camera Aperture Range	f/2.8 - f/11
Post-processing Software Supported Software	DJI Terra

## Ethics Statement

The authors state that the present work meets the ethical requirements for publication in Data in Brief. The work does not involve studies with animals and humans.

## CRediT authorship contribution statement

**Sergio Vélez:** Investigation, Visualization, Methodology, Data curation, Writing – original draft. **Mar Ariza-Sentís:** Visualization, Writing – review & editing, Methodology, Data curation. **João Valente:** Conceptualization, Supervision, Writing – review & editing.

## Data Availability

High resolution LiDAR dataset acquired using UAV (unmanned aerial vehicle) over two vineyards and two years located in `Tomiño', Pontevedra, Spain. (Original data) (Zenodo)
